# Health benefits versus economic costs: welfare analysis of energy conservation and emission reduction

**DOI:** 10.3389/fpubh.2025.1662116

**Published:** 2025-09-26

**Authors:** Hongkun Zhao, Zhuo Chen, Nan Feng

**Affiliations:** ^1^School of Economics, Liaoning University, Shenyang, China; ^2^School of Economics and Management, Inner Mongolia Normal University, Hohhot, China; ^3^School of Economics, Dongbei University of Finance and Economics, Dalian, China

**Keywords:** health effect, welfare analysis, labor market performance, energy conservation and emission reduction, a longitudinal study

## Abstract

**Introduction:**

A multidimensional and comprehensive evaluation of the impact of energy conservation and emission reduction (ECER) on residents’ health and welfare is conducive to resolving conflicts between economy and environment on a worldwide scale.

**Methods:**

Based on China’s ECER demonstration city policy, this paper uses a staggered difference-in-differences method to examine the impact of ECER on residents’ health and labor market performance, and conservatively estimates the welfare effect of ECER in conjunction with a theoretical model.

**Results:**

The results show that ECER significantly improves residents’ health, raises self-rated health (*β* = 0.06, *p* < 0.05, 95% CI = −0.17 to 0.13), reduces the probability of illness affecting work (*β* = −0.004, *p* < 0.05, 95% CI = −0.01 to 0.01), and lowers medical expenditures (*β* = −0.183, *p* < 0.05, 95% CI = −0.64 to 0.10). However, ECER negatively affects residents’ labor market performance, reducing employment status (*β* = −0.032, *p* < 0.10, 95% CI = −0.11 to 0.06) and wage (*β* = −0.055, *p* < 0.05, 95% CI = −0.23 to 0.00). Mechanism analysis suggests that ECER primarily improves health by reducing emissions of pollutants such as urban industrial wastewater, industrial sulphur dioxide, and industrial fumes and dust, and negatively influences labor market performance by promoting industrial restructuring. Heterogeneity analysis shows that there is a selection effect in the impacts, the health benefits and economic costs of ECER are mostly achieved and borne by groups in rural areas, non-provincial capitals, and those suffering from chronic diseases and not engaging in physical activity. Welfare analysis suggests that the health benefits of ECER result in higher welfare gains than the negative welfare impacts of its economic effects.

**Conclusion:**

Future policies should progressively move towards an integrated assessment of the costs and benefits of ECER, paying particular attention to welfare losses among groups that bear higher costs.

## 1 Introduction

In the process of economic development, resolving contradiction between economy and environment is a major challenge commonly faced by all countries. Numerous studies have shown that environmental pollution resulting from economic development has led to a series of severe health problems, including respiratory infections, lung cancer, and even death (1, 2). The health of the population is a fundamental component of human development, functioning both as a prerequisite and the ultimate objective of development. Nevertheless, initiatives such as Energy Conservation and Emission Reduction (ECER) demonstration city policy, which aims to regulate environmental pollution, have the potential to adversely impact employment and economic growth. A number of studies have indicated that ECER can impede enterprise output and diminish labor demand, consequently resulting in economic distortion (3, 4). It is therefore imperative that a comprehensive consideration of the health and economic effects of the implementation of ECER is given due consideration in the pursuit of sustainable development, both in China and on a global scale.

However, while ECER is essential for protecting environment and public health, its positive effects on improving local labor market have not been fully realized due to a lack of necessary investment and support. In response to the complex interplay between energy efficiency, emission reduction, health, and the resident labor market, China Ministry of Finance and the National Development and Reform Commission established 30 ECER city pilots in three batches in 2011, 2013, and 2014. The ECER demonstration cities focus on intensifying ECER efforts, advocating for greener lifestyles, promoting a green transformation of the economy, and enhancing the health and well-being of residents. Can these city pilots significantly impact resident health and labor market performance? Is there any heterogeneity in the effects of the pilot on residents’ health and labor market performance? What are the mechanisms underlying these effects? Answering these questions within the context of ECER pilot program will help provide a comprehensive understanding of the impacts of ECER on residents’ welfare.

As the largest developing country, China faces complex challenges related to health and employment (5, 6). Despite significant progress in improving healthcare resources, social security, and workers’ rights and benefits, China continues to grapple with healthcare burdens and underemployment issues, owing to the scarcity of healthcare resources and the mismatch between human resource supply and demand (7, 8). Driven by national ECER policy, government has implemented economic, legal, and administrative measures. From 2013 and 2023, China experienced an average annual economic growth of 6.1%, while achieving an average annual growth rate of 3.3% in energy consumption, with a cumulative decrease in energy intensity of 26.1%. This represents one of the fastest reductions in energy intensity globally and has contributed significantly to global ECER efforts. The implementation of ECER has generated substantial environmental and economic effects, and the resulting welfare impacts need to be urgently examined. Given its typical energy and environmental context and large population, China offers a valuable research case for exploring the causal relationship between ECER and residents’ welfare.

**Figure 1:**
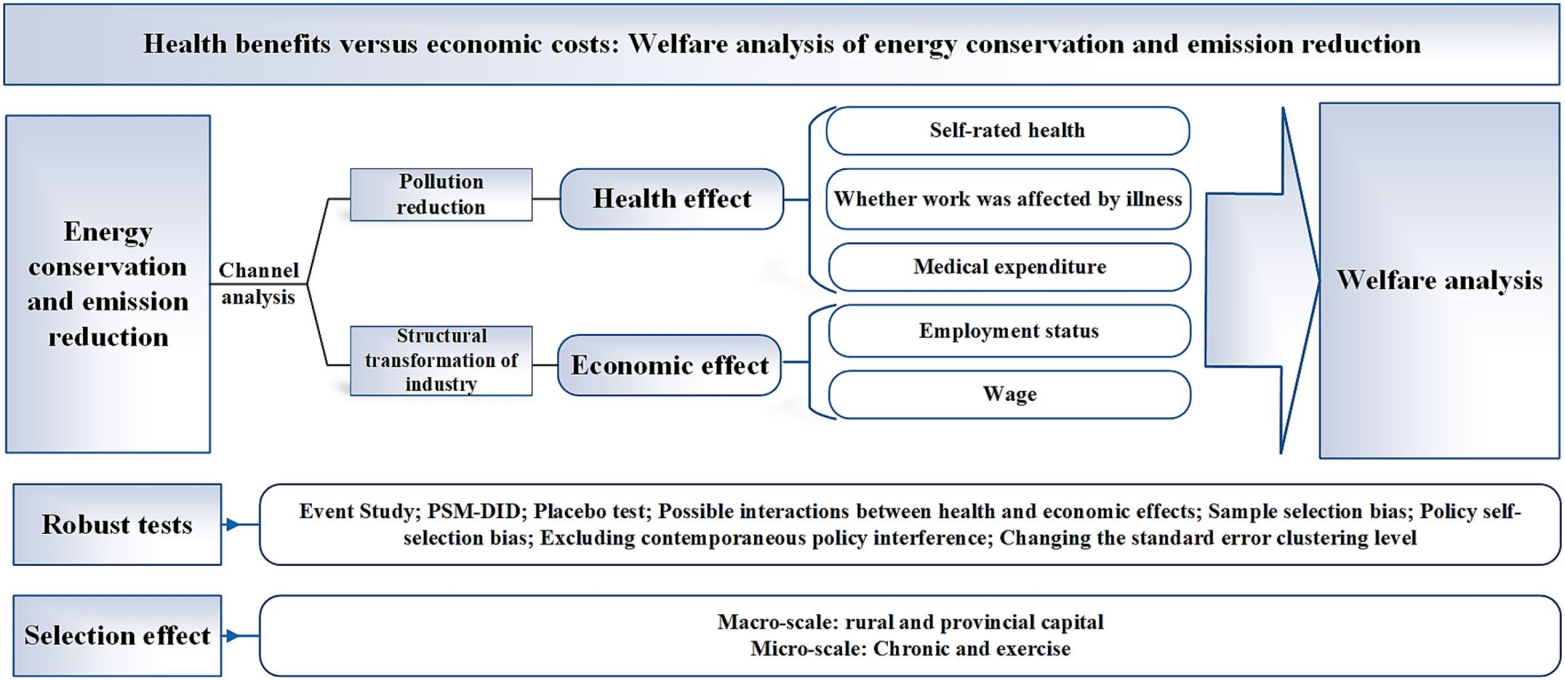
Research framework.

Based on the quasi-natural experiment of ECER pilot city policy in China, this paper employs staggered difference-in-differences method to examine the impact of ECER on residents’ health and labor market performance from residents’ perspective. Furthermore, the welfare effect of ECER is conservatively estimated by combining it with a constructed theoretical model. The conclusion remains robust after conducting various robustness tests, including event study, PSM-DID estimation, placebo tests, excluding the interaction between economic and health effects, addressing sample selection bias, self-selection bias, and contemporaneous policy disturbances, as well as altering the standard error clustering level. In terms of mechanism analysis, ECER reduces pollutant emissions, thereby improving residents’ health. Simultaneously, it promotes the transformation of industrial structure, which impacts residents’ labor market performance. Heterogeneity analysis reveals that there is a selection effect in impact, with health benefits and economic costs of ECER being predominantly experienced by groups in rural areas, non-provincial capital cities, and those suffering from chronic diseases or engaging in no exercise. Welfare analysis indicates that the health benefits of ECER outweigh its economic costs in terms of welfare increase. Research framework is shown in Figure 1.

This study makes three key contributions. First, most existing analyses on ECER have focused on the firm or regional level (9, 10). This paper complements such research by examining the impacts of ECER from an individual health perspective, providing insights into how environmental policies affect individuals directly. Second, while much of existing literature addresses the impacts of pollution, health, or the economy separately (7, 11, 12), we employ a theoretical model to estimate combined impacts of ECER on individual welfare. The model allows for a more comprehensive evaluation of ECER, enabling us to assess both health and economic effects on residents and avoid biases in estimating the overall welfare effects of pollution reduction. Third, the innovative use of exogenous policy shocks confirms that the health improvement effect of ECER, achieved through pollution reduction and industrial structure specialization, is greater than its economic distortion effect. Our findings indicate that ECER enhances the overall welfare of residents.

This paper is organized as follows. Section 2 describes ECER background. Section 3 presents theoretical model. Section 4 describes research design. Section 5 empirically analyzes economic and health effects of ECER. Section 6 conducts mechanism test and heterogeneity analysis. Section 7 further estimates welfare effects of ECER. Finally, Section 8 summarizes the study.

## 2 Policy background

China’s economy, currently in transition, is facing increasingly urgent pressure to reduce emissions, and ECER has become the inevitable choice for the sustainable development of China’s economy in the new period. In the 11th Five-Year Plan, China set “reducing energy consumption per unit of GDP by about 20%” and ‘reducing total emissions of major pollutants by 10%’ as binding targets for the first time (13). Subsequently, the 12th Five-Year Plan further proposed the target of reducing carbon dioxide emissions per unit of GDP by 17% by 2015 (14).

**Figure 2:**
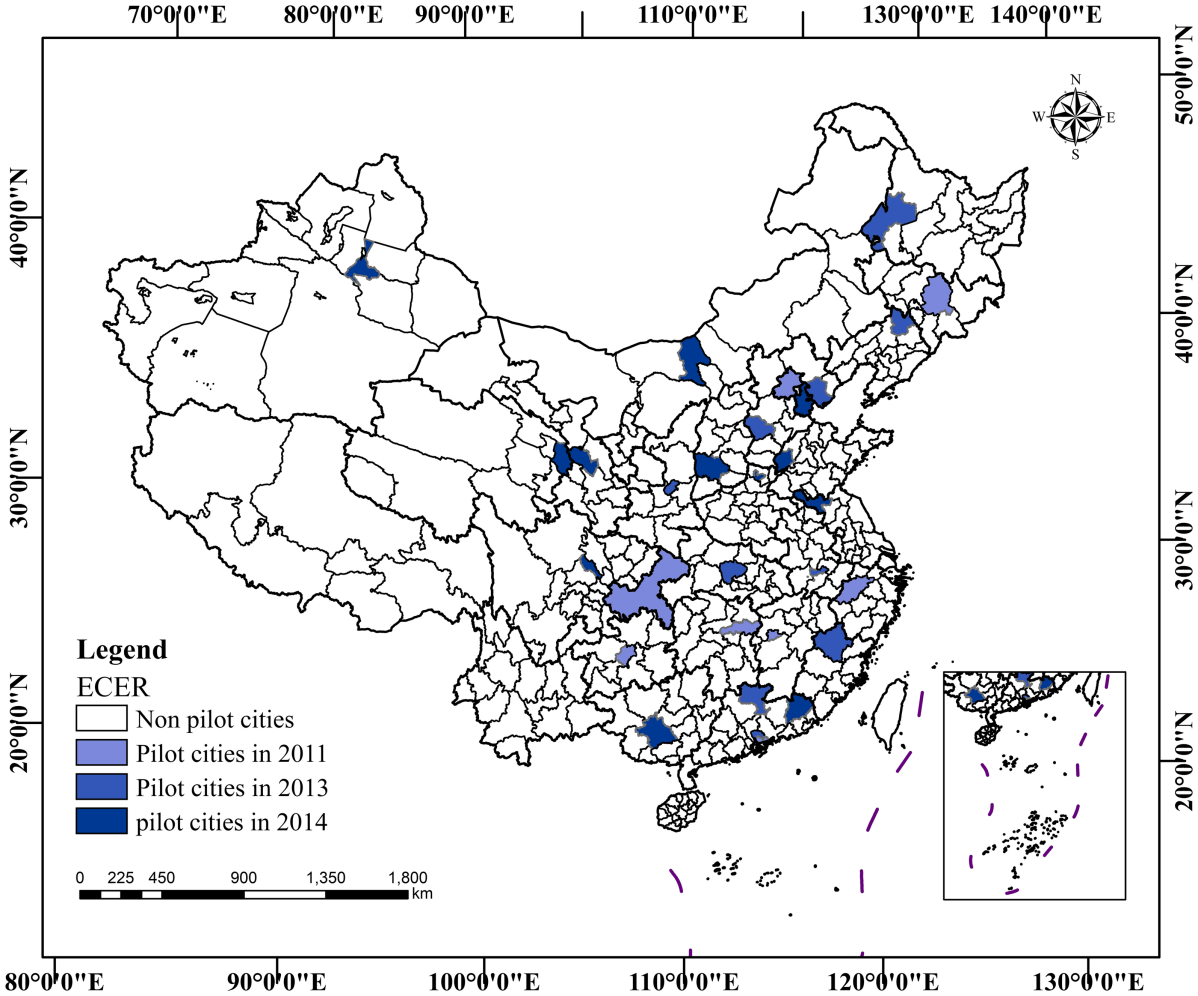
Distribution of ECER demonstration cities.

To successfully achieve these binding targets for ECER and enhance environmental sustainability, the Ministry of Finance and the National Development and Reform Commission issued the “Circular on the Comprehensive Demonstration of ECER Fiscal Efforts” in June 2011 (15). This circular identified eight cities as demonstration cities, thereby initiating the construction of these demonstration cities. Subsequently, based on summarizing the construction experience of the first batch of demonstration cities, the second and third batches of ECER demonstration lists were determined in 2013 and 2014, respectively (16). By the end of 2014, a total of 30 cities had been identified as ECER demonstration cities, as shown in Figure 2.

ECER is implemented in both industrial and domestic sectors, with the goal of promoting the efficiency of green economy and improving human environment in demonstration cities (17). The selection of demonstration cities is directly decided by central government, which largely ensures that the policy constitutes an exogenous shock. We also confirmed the exogeneity and randomness of demonstration cities selection through various robustness tests. At the same time, the selected cities differ significantly in terms of economic characteristics, city size, and demonstration content, thus making them highly representative and exemplary.

## 3 Theoretical framework

Building on Grossman’s health capital framework (18), which conceptualizes health as both a consumption and an investment good, we extend the analysis to incorporate policy-induced changes in health outcomes. Insights from Fan et al. (19) and Xie and Feng (20), which highlight the broader health and welfare implications of economic and environmental policies, further motivate the construction of our theoretical framework. Based on these foundations, we develop a model to analyze the health and economic impacts of ECER and subsequently estimate its welfare effects. Although there could theoretically be interactions between these effects, our empirical analysis does not detect any significant interaction. Consequently, our framework does not account for such interactions and is structured as follows.

Individuals are assumed to derive utility from consumption *c*, health *h* and leisure *l*, specified as Equation 1:

(1) $$U=u(c,h,l)=\ln c+h-h_{0}-\frac{l^{1+\gamma }}{1+\gamma },\gamma >0$$

Assume that health *h* is only a function of medical expenditures *M*, specific as Equation 2:

(2) $$h=h(M)\geq h_{0}=h(M_{0})$$

Let *h*_0_ represent the minimum health level required for a resident to live a healthy life, and let *M*_0_ be the minimum medical expenditure necessary to sustain this health level. While ECER may influence health through various channels beyond medical care, such as other forms of health investment, this framework focuses primarily on medical expenditures, which have the most significant impact on health outcomes. It is acknowledged that reductions in pollution have beneficial effects on health, so other unconsidered factors in health function may also have positive effects on health. Consequently, the exclusion of these factors may result in an underestimation of the health benefits of ECER, thereby rendering welfare estimates more conservative.

Assume that income *I* is used only for *c* and *M*, specific as Equation 3:

(3) c+M<I=wl

where *w* denotes wage. *γ* denotes the inverse of Frisch labor supply elasticity. The Frisch labor supply is concerned with keeping the marginal utility of wealth constant, and the values of its elasticity are all greater than or equal to 0 less than 1 (21). Regarding employment and labor time, since the demand side of the China’s labor market has strong bargaining power, while employees have limited negotiating leverage and must rely on employment to meet their basic needs, the level of employment and hours worked are predominantly shaped by demand (19, 22).

So, individual utility function is given by Equation 4:

(4) $$U=\ln(\mathrm{wl}-M_{0})-\frac{\mathrm{wl}}{\mathrm{wl}-M_{0}}\times \frac{1}{1+\gamma }$$

However, under the effect of ECER policy, ECER *E* may affect individual’s utility by affecting *h*, *l*, and *w*, as outlined below.

Regarding health outcomes, ECER can improve residents’ health by addressing environmental factors, particularly pollution reduction. Policies such as emission fees or green lifestyle promotion implemented under ECER can significantly lower pollution levels, thereby enhancing public health (23).

At the aspect of economic effect, ECER can influence employment and wages through two primary mechanisms: increased costs and innovation. Stricter ECER regulations raise production costs by requiring companies to use cleaner but more expensive energy sources, invest in pollution control equipment. These changes can reduce the demand for various inputs, including labor, potentially leading to lower employment and wage levels (3). Conversely, ECER policies encourage firms to innovate, developing environmentally friendly products and technologies. This innovation not only boosts demand for skilled workers but also enhances firm productivity and profitability, thereby increasing the demand for both skilled and unskilled labor, which can positively impact individuals’ economic status (24).

Considering aforementioned effects of ECER on individual utility, so optimization problem is specified as Equation 5:

(5) $$U=\max u(c,h,l)=\max_{c,h,l}\ln c+h-h(M_{0})-\frac{l^{1+\gamma }}{1+\gamma }$$

s.t.c+M≤I(E)=w(E)l(E)

$$h=h(M)\geq h(M_{0})$$

$$\frac{\partial M_{0}}{\partial E}<0$$

In this case, ECER may be beneficial to improve individuals’ health by reducing pollution, so the relationship between *M*_0_ and *E* is assumed to be negative (∂*M*_0_/∂*E* < 0).

Solving above optimization problem according to the first order conditions can be obtained as Equation 6:

(6) $$\left\{ \begin{array}{c} c=w(E)l(E)-M_{0}(E)\\ \begin{array}{c} M=M_{0}(E)\\ h(M)=h(M_{0})\\ w(E)=l{\left(E\right)}^{\gamma }\times c \end{array} \end{array}\right.$$

Thus, the connection between ECER and individual utility is described as Equation 7:

(7) $$\begin{array}{l} U=V(I,M_{0})=\ln(w(E)l(E)-M_{0}(E))\\ -\frac{w(E)l(E)}{w(E)l(E)-M_{0}(E)}\times \frac{1}{1+\gamma } \end{array}$$

Equation 7 synthesizes the overall welfare effects of ECER, encompassing both health and economic dimensions. While ECER can enhance individual welfare by improving health and lowering healthcare costs, it can also diminish welfare by decreasing employment and wage. It is important to note that our welfare framework defines health solely as a function of medical expenditures, without explicitly incorporating the direct adverse effects of environmental pollution exposure. Consequently, the estimated welfare gains are likely to be conservative, as they may underestimate the full health benefits derived from reductions in pollution.

Theoretical framework outlined above suggests that identifying the impact of ECER on welfare requires testing the following two hypotheses:

*Hypothesis 1*: ECER is conducive to the improvement of individual health, i.e., the health improvement effect of ECER.

*Hypothesis 2*: ECER leads to lower individual employment and income, i.e., the economic distortion effect of ECER.

Then, the impact of ECER on individual welfare is then conservatively estimated based on the results obtained and in conjunction with the welfare analysis framework. This can be achieved by deriving Equation 7 with respect to ECER:

(8) $$\frac{d\ln U}{\mathrm{dE}}=\frac{1}{U}\times \frac{\mathrm{dU}}{\mathrm{dE}}$$

(9) $$\begin{array}{l} \frac{\mathrm{dU}}{\mathrm{dE}}=-\frac{M_{0}}{I-M_{0}}(1+\frac{1}{1+\gamma }\times \frac{I}{I-M_{0}})\times \frac{d\ln M_{0}}{\mathrm{dE}}\\ +\frac{w}{I-M_{0}}(1+\frac{1}{1+\gamma }\times \frac{M_{0}}{I-M_{0}})\times \frac{\mathrm{dl}}{\mathrm{dE}} \end{array}$$

Where *d*ln*U*/*dE* measures the impact of ECER on individual welfare. The first term to the right of Equation 9 indicates the impact of health effect of ECER on individual welfare, which can be calculated based on sample means and regression results in Table 1, columns 5 and 6 from empirical section. The second term to the right of Equation 9 represents the impact of economic effect of ECER on individual welfare, which can be calculated based on sample means and regression results in columns 3 and 4 of Table 2 from empirical section.

**Table 1:** Health effects of ECER.

Variable	*SRH*	*SRH*	*TWDI*	*TWDI*	*log*(*ME*)	*log*(*ME*)
	(1)	(2)	(3)	(4)	(5)	(6)
*ECER*	0.066**	0.060**	−0.004***	−0.004**	−0.146*	−0.183**
	(0.028)	(0.030)	(0.001)	(0.002)	(0.087)	(0.088)
Individual characteristic	No	Yes	No	Yes	No	Yes
Household characteristics	No	Yes	No	Yes	No	Yes
Urban characteristics	No	Yes	No	Yes	No	Yes
Individual fixed effect	Yes	Yes	Yes	Yes	Yes	Yes
Year fixed effect	Yes	Yes	Yes	Yes	Yes	Yes
Urban fixed effect	Yes	Yes	Yes	Yes	Yes	Yes
*Obs*	78,575	68,575	78,575	68,575	46,993	43,314
*R* ^2^	0.589	0.581	0.572	0.561	0.496	0.493

*, **, and *** denote significance at 10, 5, and 1% levels. Robust standard errors are in parentheses. The same below.

**Table 2:** Economic effects of ECER.

Variable	*ES*	*ES*	*log*(*Wage*)	*log*(*Wage*)
	(1)	(2)	(3)	(4)
*ECER*	−0.031**	−0.032*	−0.061**	−0.055**
	(0.016)	(0.018)	(0.026)	(0.027)
Individual characteristic	No	Yes	No	Yes
Household characteristics	No	Yes	No	Yes
Urban characteristics	No	Yes	No	Yes
Individual fixed effect	Yes	Yes	Yes	Yes
Year fixed effect	Yes	Yes	Yes	Yes
Urban fixed effect	Yes	Yes	Yes	Yes
*Obs*	58,628	51,544	60,313	53,950
*R* ^2^	0.711	0.702	0.344	0.334

## 4 Research design

### 4.1 Samples and data

We employ data from CHARLS in 2011, 2013, 2015, 2018, and 2020 as research sample, focusing on prefecture-level regions. These specific years were chosen because they correspond to the official survey waves of CHARLS, which are conducted every 2–3 years and represent the only periods for which microdata is available. CHARLS is a research project sponsored by National Development Research Institute of Peking University and implemented by Center for Chinese Social Science Survey of Peking University. CHARLS interviewed and recorded microdata on individuals, households, and communities of middle-aged and older adults aged 45 and above. Data were collected from households in 150 counties, spanning 450 village-level administrative districts nationwide. We matched ECER demonstration cities with individuals’ data in CHARLS. A valid sample of 96,531 individuals was obtained from 115 prefecture-level cities, covering 12,408 households and residents in 450 survey areas nationwide. Control variables for prefecture level were obtained from China Urban Yearbook and provincial and municipal statistical yearbooks. Descriptive statistics of variables are shown in Table 3.

**Table 3:** Descriptive statistics

Variable	Define	Observations	Mean	Standard deviation	Min	Max
SRH	Self-rated health	90,716	3.045	0.986	1.000	5.000
TWDI	Impact on work due to illness	90,716	0.103	0.052	0.000	1.000
ME	Medical expenditure	91,612	3642.674	15201.219	0.000	1200000.000
ES	Employment status	72,076	0.697	0.459	0.000	1.000
Wage	Annual wages	68,358	28313.402	13760.457	0.000	262500.000
Age	Age	96,339	60.680	10.490	45.000	118.000
Edu	Educational level	96,489	2.019	1.056	1.000	4.000
Gender	Gender	82,101	0.465	0.499	0.000	1.000
Rural	Urban or rural	96,628	0.596	0.491	0.000	1.000
Marry	Marital status	96,531	0.860	0.347	0.000	1.000
Retire	Retired or not	94,845	0.145	0.352	0.000	1.000
Exercise	Exercise or not	61,927	0.894	0.308	0.000	1.000
Chronic	Whether you have a chronic disease	92,276	0.759	0.428	0.000	1.000
HB	Number of hospital beds	86,460	27798.617	24557.251	3646.000	174957.000
GDP	Per capita gross regional product	86,353	49529.518	31107.042	6916.000	467749.000

### 4.2 Definitions and variables

#### 4.2.1 Explained variable

Explained variables in this study are categorized into health indicators and economic indicators. The health indicators use three indicators, self-rated health (*SRH*), whether work was affected by illness in the last year (*TWDI*), and medical expenditure in the last year (*ME*), to measure the health status of individuals (7, 25). Among them, the *SRH* uses a five-point Likert scale score evaluation system to measure the individual’s health level from low to high (26). While *SRH* is a widely adopted indicator in health economics literatures, it may be subject to individual cognitive biases. To mitigate this limitation, we complement *SRH* with two relatively more objective measures, *TWDI* and *ME*, which provide behavioral and financial perspectives on individual health outcomes. For the economic effect, the main focus is on the individual’s labor market performance, so the economic indicators use employment status (*ES*) and last year’s wage income (*Wage*) as measures of labor market performance (27).

#### 4.2.2 Explanatory variable

Core explanatory variable is the dummy variable for ECER demonstration policy. As three batches of ECER have been implemented and there are duplicate cities in the three pilot batches, the time of the first pilot batch is used as the time point. And referring to the theory of health needs, we select control variables that may affect health and labor market performance from three dimensions: individual, household and city (18). Control variables at individual or household level included age, gender, marital status, education, whether retired or not, and urban or rural, while control variables at the regional level included per capita gross regional product and the number of hospital beds. A one period lag is applied to city-level variables.

### 4.3 Empirical strategy

#### 4.3.1 Baseline model

ECER demonstration policy provides a good natural experimental environment for how ECER affects residents’ welfare. Considering that ECER demonstration cities are implemented in batches, this paper adopts staggered difference-in-differences method to study ECER’s impact on residents’ welfare, and econometric model is set as follows:

(10) $$Y_{\text{ijct}}=\beta_{0}+\beta_{1}\mathrm{ECE}R_{\mathrm{ct}}+\text{γContro}l_{\text{ijct}}+\eta_{i}+\omega_{j}+\nu_{c}+\lambda_{t}+\varepsilon_{\text{ijct}}$$

where *i*, *j*, *c*, and *t* denote individual, household, city, and year, respectively; *Y_ijct_* is the dependent variable, containing health and economic indicators; *ECER_ct_* represents whether city *c* was a demonstration in year *t*; *Control_ijct_* denotes individual characteristics, household characteristics, or city characteristics, including age, gender, education, marriage, whether retired, urban/rural, per capita GDP and number of hospital beds; *η_i_*, *ω_j_*, and *υ_c_* denote individual fixed effect, household fixed effect, and city fixed effect, respectively, controlling for factors that do not vary over time at individual, household, or city level; *λ_t_* is a time fixed effect, controlling for factors that vary only over time; *ε_ijct_* is a random disturbance term, and to deal with autocorrelation, the clustering at the household level is used with robust standard errors.

#### 4.3.2 Event study

To alleviate concerns about parallel trends, we employ event study for validation (28). We test whether time trends in health or economic levels remain consistent between treatment and control before policy is implemented. We also observe the dynamic effects that occur after policy implementation (29). The construction of event study method is described in Equation 11.

(11) $$\begin{array}{l} Y_{\text{ijct}}=\alpha +\sum_{k=-2}^{k=3}\beta_{k}1\{t=t_{i}^{*}+k\}\times {\text{ECER}}_{c}+\text{γContro}l_{\text{ijct}}\\ +\eta_{i}+\omega_{j}+\nu_{c}+\lambda_{t}+\varepsilon_{\text{ijct}} \end{array}$$

where *Y_ijct_* is the outcome variable for individual *i* of household *j* in city *c* in year *t*; *ECER_c_* is a dummy equal to 1 if city *c* is an ECER demonstration city; 1{*t* = t_i_* + *k*} is an event dummy variable; it is the year in which individual *i* experienced the ECER event; *Control_c_* is a control variable, which is the same as the above variables, and includes individual, household, and key characteristics at the city level. We control for time fixed effect *λ_t_*, individual fixed effect *η_i_*, household fixed effect *ω_j_*, and city fixed effects *υ_c_*. We drop dummy variables associated with the year prior to the ECER city pilot event so that *βk* specifically captures the change in the treatment outcome variable, *Y_ijct_*, relative to the baseline difference observed at *k* = −1, compared to its counterfactual counterpart. Standard errors are clustered at household level.

#### 4.3.3 Heckman two-step method

Because the characteristics of cities classified as ECER pilots differ from those not classified as ECER pilots, the residents of the pilot cities may themselves exhibit high or low levels of the dependent variable (e.g., health status or labor market performance), or the pilot cities themselves may have strong adaptive capacity for energy efficiency and emission reduction policies. Given the potential for self-selection bias, we use the Heckman two-stage method to address it (30).

In the first stage model, the dependent variable “whether the city is a ECER demonstration city” is dichotomized, and comprises all control variables used in the second stage model. In addition, we use city electricity consumption as an instrumental variable to satisfy the exclusion restriction. Electricity consumption is one of the most important indicators of cities’ energy consumption, and cities with high electricity consumption have more potential and space for ECER. The implementation of ECER measures has been demonstrated to result in substantial reductions in energy consumption and emission levels. Consequently, cities with high electricity consumption are more likely to be selected as demonstration cities. The first stage model and IMP are shown in Equations 12, 13, respectively.

(12) $$\mathrm{ECE}R_{\mathrm{ct}}^{*}=\alpha_{0}X_{\mathrm{ct}}+\alpha_{1}\text{Contro}l_{\mathrm{ct}}+\varepsilon_{\mathrm{ct}}$$

where $\mathrm{ECE}R_{\mathrm{ct}}^{*}$ is whether city *c* is a ECER demonstration city in year *t*, *X_ct_* is electricity consumption of city *c* in year *t*, and *Control_ct_* indicates control variables.

The predicted individual probabilities from the first stage model are combined into an additional explanatory variable, Inverse Mills ration (*IMR*), which corrects for the self-selection problem along with other variables such as control variables. *IMR* is calculated as:

(13) $$\mathrm{IMR}=E(u|D)=\{\begin{array}{c} \frac{\varphi ({\hat{\alpha }}_{0}^{\prime }Z+{\hat{\alpha }}_{1}^{\prime }X)}{\Phi ({\hat{\alpha }}_{0}^{\prime }Z+{\hat{\alpha }}_{1}^{\prime }X)}\;\mathrm{ifD}=1\\ -\frac{\varphi ({\hat{\alpha }}_{0}^{\prime }Z+{\hat{\alpha }}_{1}^{\prime }X)}{\left(1-\Phi ({\hat{\alpha }}_{0}^{\prime }Z+{\hat{\alpha }}_{1}^{\prime }X)\right)}\;\text{if}\;D=0 \end{array}$$

where *φ*( ) represents regular conditional probability density function and *Φ*( ) is the cumulative distribution function of standard normal random variables. Subsequently, the calculated *IMR* is added to the baseline regression model Equation 10 for regression. Ultimately, the econometric model of Heckman two-step method is presented as Equation 14.

(14) $$\begin{array}{l} Y_{\text{ijct}}=\beta_{0}+\beta_{1}\mathrm{ECE}R_{\mathrm{ct}}+\mathrm{IM}R_{\text{ijct}}+\text{γContro}l_{\text{ijct}}\\ +\eta_{i}+\omega_{j}+\nu_{c}+\lambda_{t}+\varepsilon_{\text{ijct}} \end{array}$$

Where *IMR_ijct_* is Inverse Mills ration.

## 5 Evaluation of health and economic effects of ECER

### 5.1 Baseline estimation

#### 5.1.1 Health effect of ECER

Health effect of ECER as shown in Table 1. Health effect of ECER estimated here have excluded cases where ECER affects health through economic channels. From columns 1, 3 and 5, it can be found that ECER improves residents’ health, significantly reduces the probability of residents’ work being affected by illness, and also significantly reduces individuals’ healthcare expenditures, thereby effectively improving residents’ health. To further exclude the influence of some unobservable factors on results, columns 2, 4 and 6 additionally control for individual, household and urban characteristics. It can be found that results are robust under more stringent model settings. In regard to magnitude of impact, according to results in columns 2, 4 and 6, it can be seen that ECER enhances the subjective evaluation of personal health by 6.0%, and leads to a reduction in probability of an individual’s work being affected by illness by 0.4% and decreases medical expenditures by 18.3%, respectively, respectively. The above analysis indicates that ECER exerts a significant positive effect on health, which is consistent with hypothesis 1. These findings align with previous empirical evidence indicating that reductions in pollution and enhanced environmental regulations significantly improve health outcomes and reduce medical expenditure (31–33). These consistency with existing literatures further strengthens the validity of our findings.

#### 5.1.2 Economic effect of ECER

Table 2 reports impact of ECER on residents’ employment status and wage. From columns 1 to 4, it can be seen that the impact of ECER on residents’ employment status and wag is significantly negative. Specifically, column 2 shows a 3.2% reduction in employment status, while column 4 indicates a 5.5% reduction in wage. The results indicate that ECER exerts a significant negative effect on residents’ labor market performance, which proves hypothesis 2.

#### 5.1.3 Dynamic trends of ECER impacts on health and economy

**Figure 3:**
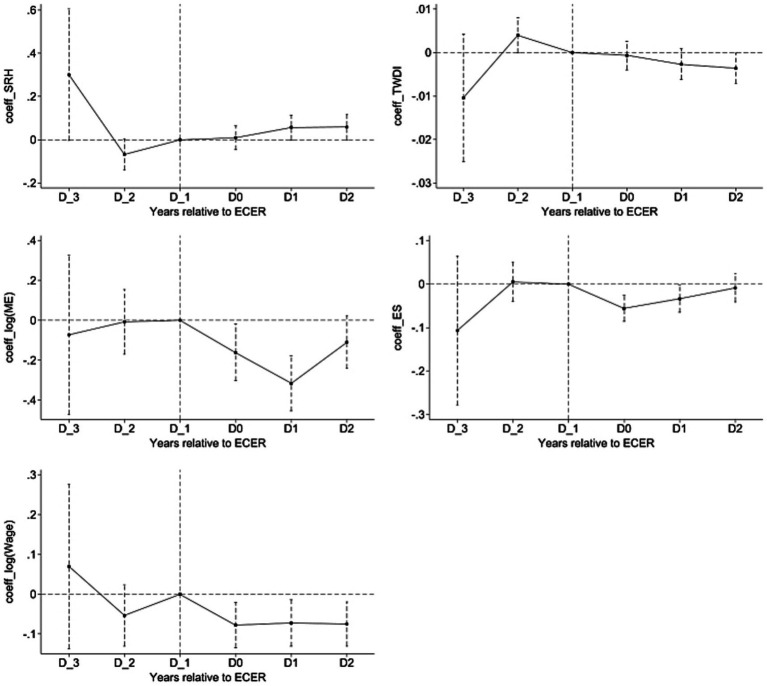
Dynamic trends of ECER impacts on health and economy.

Figure 3 reports the estimated coefficients βk for health and economic effect of ECER under event study method. Vertical line indicates year prior to the implementation of ECER policy. It can be observed that, regarding both health effect and economic effect, estimated coefficients are not significant and show no obvious trend before the implementation of ECER city pilot. None of estimated values exhibit a more obvious trend during this period, which suggests that the parallel trend assumption is satisfied. After the implementation of ECER policy, absolute values of estimated coefficients changed significantly: *SRH* showed an upward trend, while *TEDI*, log(*ME*), *ES*, and log(*Wage*) showed downward trends. This indicates that residuals’ health and economic status changed significantly after the implementation of ECER demonstration policy, supporting results of baseline regression.

### 5.2 Robustness test

#### 5.2.1 PSM-DID estimation

Government’s selection of ECER demonstration cities may be non-random, potentially biasing the estimated parameters of baseline regression and failing to accurately reflect true impact of ECER on residents’ health or the economy. To reduce this sample selection bias, this study employs PSM-DID method to test baseline results (34). PSM provides a variety of matching methods, including nearest-neighbor, radius, and kernel matching. Due to the large sample size and in pursuit of robust results, this study chooses the nearest-neighbor matching method. Specifically, control variables are first selected as matching variables to screen the study sample. This is followed by 1:4 nearest-neighbor matching, and then the regression estimation is re-run using matched samples. Table 4 reports test results. After addressing the sample selection problem, the positivity and significance of estimated coefficients of variables remain consistent with the findings from baseline regression.

**Table 4:** PSM-DID estimation.

Variable	Health effect			Economic effect	
	*SRH*	*TWDI*	*log*(*ME*)	*ES*	*log*(*Wage*)
*ECER*	0.089**	−0.005***	−0.342***	−0.051***	−0.070*
	(0.034)	(0.002)	(0.101)	(0.020)	(0.041)
Individual characteristic	Yes	Yes	Yes	Yes	Yes
Household characteristics	Yes	Yes	Yes	Yes	Yes
Urban characteristics	Yes	Yes	Yes	Yes	Yes
Individual fixed effect	Yes	Yes	Yes	Yes	Yes
Year fixed effect	Yes	Yes	Yes	Yes	Yes
Urban fixed effect	Yes	Yes	Yes	Yes	Yes
*Obs*	15,568	15,568	8,907	11,821	4,387
*R* ^2^	0.656	0.637	0.550	0.551	0.419

#### 5.2.2 Placebo test

**Figure 4:**
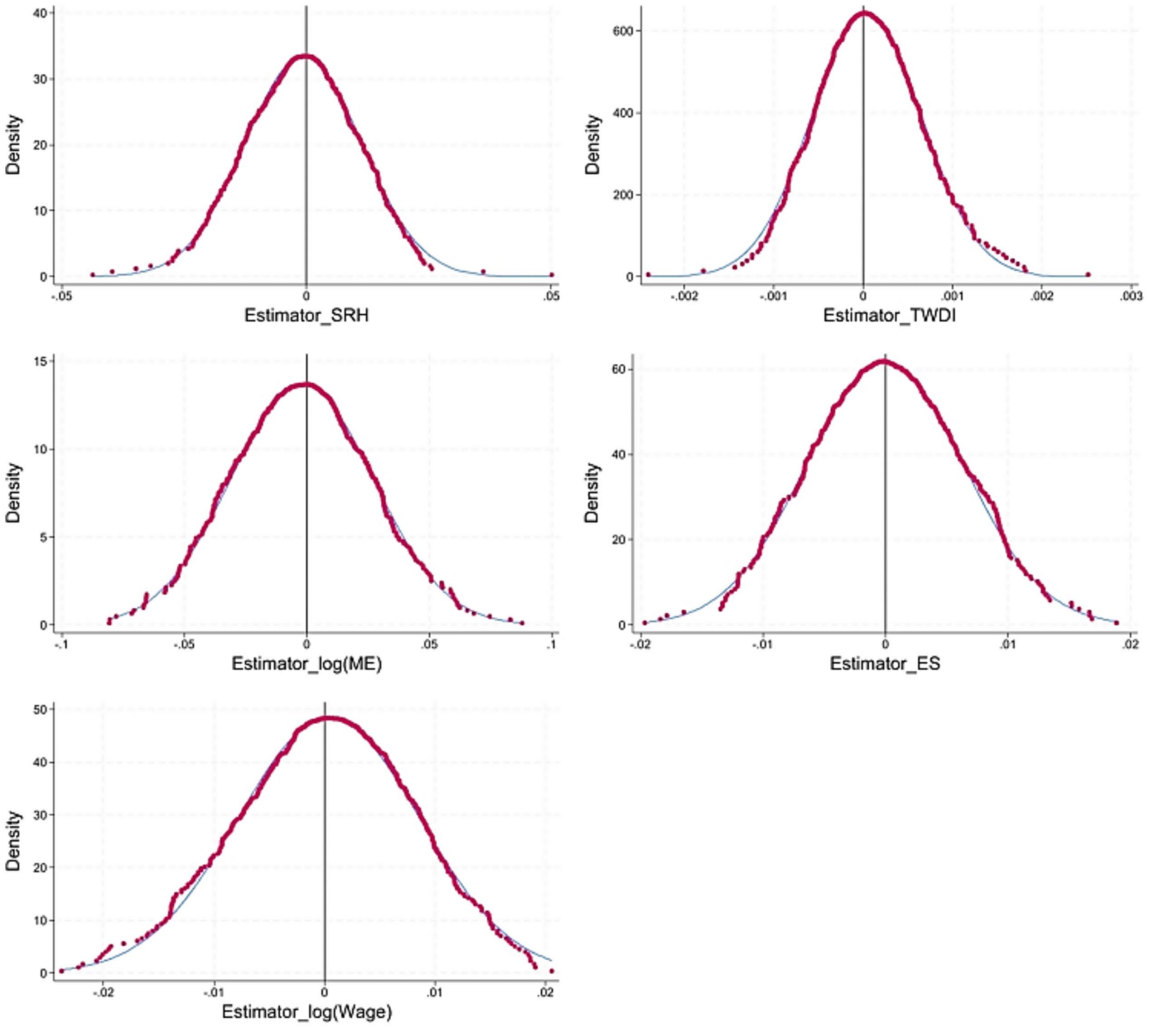
Placebo test.

To exclude non-randomness of ECER demonstration cities and the possible impact of other policies on conclusions, we construct pseudo-policy dummy variables for a random sample of 500 iterations and rerun the regression estimation using Equation 8. If the estimate of policy effect is zero after dropping the policy effect, it indicates that other missing characteristics have little impact on results. Figure 4 reports the estimation results and *p*-value distribution of 500 regressions using pseudo-dummy variables. The estimated coefficients are observed to be concentrated around zero, with no overlap observed when compared to the coefficients estimated in baseline regression. This suggests that baseline regression is robust and that the conclusions are hardly affected by other non-stochastic factors.

#### 5.2.3 Possible interactions between health and economic effects

Table 5 compares the effects of ECER on health, with and without controlling for economic effect. Specifically, columns 1, 3 and 5 report results without controlling for economic effect, while columns 2, 4 and 6 include these controls. It can be observed that controlling for employment and income has almost no effect on the estimated health outcomes, suggesting that the impact of ECER on health is largely independent of economic channels. In other words, the economic effect of ECER do not significantly influence its health effect.

**Table 5:** Impact of ECER’s economic effect on ECER’s health effect.

Variable	*SRH*	*SRH*	*TWDI*	*TWDI*	*log*(*ME*)	*log*(*ME*)
	(1)	(2)	(3)	(4)	(5)	(6)
*ECER*	0.060**	0.061*	−0.004**	−0.003*	−0.183**	−0.134*
	(0.030)	(0.036)	(0.002)	(0.003)	(0.088)	(0.075)
Economic effect	No	Yes	No	Yes	No	Yes
Individual characteristic	Yes	Yes	Yes	Yes	Yes	Yes
Household characteristics	Yes	Yes	Yes	Yes	Yes	Yes
Urban characteristics	Yes	Yes	Yes	Yes	Yes	Yes
Individual fixed effect	Yes	Yes	Yes	Yes	Yes	Yes
Year fixed effect	Yes	Yes	Yes	Yes	Yes	Yes
Urban fixed effect	Yes	Yes	Yes	Yes	Yes	Yes
*95% CI*	(−0.15 to 0.12)	(−0.17 to 0.13)	(−0.01 to 0.01)	(−0.01 to 0.01)	(−0.69 to 0.03)	(−0.64 to 0.10)
*Obs*	68,575	53,411	68,575	53,411	43,314	36,146
*R* ^2^	0.581	0.663	0.561	0.650	0.493	0.522

Similarly, Table 6 examines the impact of ECER on economic outcomes, with and without controlling for health effect. Columns 1 and 3 report results without controlling for health effect, while columns 2 and 4 include these controls. Results show that controlling for health has almost no effect on the estimated economic outcomes, indicating that impact of ECER on labor market performance is largely independent of health channels. In other words, the health effect of ECER do not play a significant role in its economic effect. These findings prove that there is no significant interaction between health and economic effects of ECER.

**Table 6:** Impact of ECER’s health effect on ECER’s economic effect.

Variable	*ES*	*ES*	*log*(*Wage*)	*log*(*Wage*)
	(1)	(2)	(3)	(4)
*ECER*	−0.032*	−0.031*	−0.055**	−0.051**
	(0.018)	(0.019)	(0.027)	(0.022)
Health effect	No	Yes	No	Yes
Individual characteristic	Yes	Yes	Yes	Yes
Household characteristics	Yes	Yes	Yes	Yes
Urban characteristics	Yes	Yes	Yes	Yes
Individual fixed effect	Yes	Yes	Yes	Yes
Year fixed effect	Yes	Yes	Yes	Yes
Urban fixed effect	Yes	Yes	Yes	Yes
*95% CI*	(−0.09 to 0.05)	(−0.11 to 0.06)	(−0.22 to 0.00)	(−0.23 to 0.00)
*Obs*	51,544	47,059	53,950	50,704
*R* ^2^	0.702	0.705	0.334	0.339

#### 5.2.4 Sample selection bias

First, as one of the most economically developed regions in China, the Yangtze River Delta region has significant differences in its industrial structure and energy demand compared to other regions. These differences may mask or amplify the actual impacts of ECER city pilots (35). Second, the YRD region is characterized by faster economic development, higher energy demand, more mature energy infrastructure, and a greater likelihood of implementing energy transitions and developing economies of scale. These factors have the capacity to influence overall assessment of health and economic effects of ECER. Therefore, to eliminate potential interference, we exclude the region from sample. Results in Table 7 show that exclusion of Yangtze River Delta region did not substantially affect estimation results.

**Table 7:** Results of sample selection bias test.

Variable	Health effect			Economic effect	
	*SRH*	*TWDI*	*log*(*ME*)	*ES*	*log*(*Wage*)
*ECER*	0.060**	−0.004**	−0.182**	−0.032*	−0.055**
	(0.031)	(0.002)	(0.088)	(0.018)	(0.027)
Individual characteristic	Yes	Yes	Yes	Yes	Yes
Household characteristics	Yes	Yes	Yes	Yes	Yes
Urban characteristics	Yes	Yes	Yes	Yes	Yes
Individual fixed effect	Yes	Yes	Yes	Yes	Yes
Year fixed effect	Yes	Yes	Yes	Yes	Yes
Urban fixed effect	Yes	Yes	Yes	Yes	Yes
*Obs*	Yes	Yes	Yes	Yes	Yes
*R* ^2^	0.540	0.561	0.493	0.702	0.334

#### 5.2.5 Policy self-selection bias

When analyzing the health or economic effect of ECER, it is imperative to consider the possibility of self-selection bias. Since cities’ characteristics classified as ECER pilot cities differ from those not classified as such, residents in pilot cities may inherently have higher or lower values in the dependent variable, or these cities may possess a stronger adaptive capacity to ECER policies. These factors can introduce bias into estimation results. To address this issue, this section employs Heckman two-step method to correct for possible sample selection bias encountered during previous baseline estimation.

Table 8 reports the main regression results of Heckman two-step method. In the first stage, urban electricity consumption is included to determine its impact on the designation of ECER city pilots. The results indicate that urban electricity consumption influences the delineation of ECER city pilots. The LR chi^2^ of the first stage result is 6656.34, which justifies the exclusion restriction. The second-stage results show that findings from Heckman two-step model are consistent with those of baseline regression.

**Table 8:** Heckman two-step method.

Variable	Selection equation	Health effect			Economic effect	
		*SRH*	*TWDI*	*log*(*ME*)	*ES*	*log*(*Wage*)
*ECER*		0.051*	−0.003**	−0.172**	−0.039**	−0.053*
		(0.031)	(0.002)	(0.090)	(0.019)	(0.028)
*Electronic*	0.006***					
	(0.001)					
*IMR*		−0.033	0.001	0.163	−0.186***	0.003
		(0.040)	(0.002)	(0.102)	(0.019)	(0.028)
Individual characteristic	Yes	Yes	Yes	Yes	Yes	Yes
Household characteristics	Yes	Yes	Yes	Yes	Yes	Yes
Urban characteristics	Yes	Yes	Yes	Yes	Yes	Yes
Individual fixed effect	Yes	Yes	Yes	Yes	Yes	Yes
Year fixed effect	No	Yes	Yes	Yes	Yes	Yes
Urban fixed effect	No	Yes	Yes	Yes	Yes	Yes
*Obs*	71,377	67,118	67,118	41,952	50,494	52,184
*R* ^2^	0.145	0.586	0.569	0.499	0.706	0.339
*LR chi^2^ (9)*	6656.34					

#### 5.2.6 Excluding contemporaneous policy interference

During the study period, other policies may affect residents’ health and labor market performance, potentially interfering with the effects of the ECER city pilot policy. Therefore, three policies that significantly impact these areas were selected for this study: Panel A: Smart city pilot policy; Panel B: Low carbon pilot policy; Panel C: Carbon-peaking pilot projects. Each of these policies has been identified as having a significant impact on public health and labor market performance. These policies are constructed as dummy variables and included in the baseline regression equation along with the ECER city pilot policy to capture the net effect after controlling for the effects of the other policies. Table 9 reports the regression results. Signs and significance of ECER coefficients remain consistent with those from baseline regression even after including the other policies. Even after controlling for other policies, ECER continues to play a significant role in influencing public health and labor market performance.

**Table 9:** Excluding contemporaneous policy interference.

Variable	Health effect			Economic effect	
	*SRH*	*TWDI*	*log*(*ME*)	*ES*	*log*(*Wage*)
Panel A: *Smart city pilot policy*					
*ECER*	0.062**	−0.004**	−0.168*	−0.038**	−0.054*
	(0.031)	(0.002)	(0.088)	(0.018)	(0.028)
*SCPP*	−0.008	0.001	−0.080*	0.024**	−0.008
	(0.016)	(0.001)	(0.046)	(0.018)	(0.014)
*Obs*	68,575	68,575	43,314	51,544	53,950
*R* ^2^	0.581	0.561	0.493	0.702	0.334
Panel B: *Low carbon pilot policy*					
*ECER*	0.060**	−0.004**	−0.176**	−0.032*	−0.053*
	(0.031)	(0.002)	(0.088)	(0.018)	(0.027)
*LCPP*	0.001	−0.000	−0.072	0.018*	−0.032**
	(0.016)	(0.001)	(0.044)	(0.010)	(0.014)
*Obs*	68,575	68,575	43,314	51,544	53,950
*R* ^2^	0.581	0.561	0.493	0.702	0.334
Panel C: *Carbon-peaking pilot projects*					
*ECER*	0.061**	−0.004**	−0.198**	−0.029*	−0.055**
	(0.031)	(0.002)	(0.088)	(0.018)	(0.028)
*CPPP*	−0.003	0.000	0.046	−0.012*	0.001
	0.012	(0.001)	(0.037)	(0.007)	(0.011)
*Obs*	68,575	68,575	43,314	51,544	53,950
*R* ^2^	0.581	0.561	0.493	0.702	0.334
Individual characteristic	Yes	Yes	Yes	Yes	Yes
Household characteristics	Yes	Yes	Yes	Yes	Yes
Urban characteristics	Yes	Yes	Yes	Yes	Yes
Individual fixed effect	Yes	Yes	Yes	Yes	Yes
Year fixed effect	Yes	Yes	Yes	Yes	Yes
Urban fixed effect	Yes	Yes	Yes	Yes	Yes

#### 5.2.7 Changing the standard error clustering level

In the baseline regression, this study clusters standard errors at household level to explore differences at that level. Considering that the policy effects of ECER may be more macro and exhibit homogeneity across all individuals within the same community, we also cluster standard errors at community level to examine policy at a higher level of aggregation. Results presented in Table 10 demonstrate that, with the exception of employment status, all variables maintain their statistical significance, which is consistent with findings from baseline regression. The robustness of results is maintained even when clustering at higher levels, such as community level.

**Table 10:** Changing the standard error clustering level (*community*).

Variable	Health effect			Economic effect	
	*SRH*	*TWDI*	*log*(*ME*)	*ES*	*log*(*Wage*)
*ECER*	0.060*	−0.004**	−0.183*	−0.031	−0.055*
	(0.036)	(0.002)	(0.097)	(0.026)	(0.028)
Individual characteristic	Yes	Yes	Yes	Yes	Yes
Household characteristics	Yes	Yes	Yes	Yes	Yes
Urban characteristics	Yes	Yes	Yes	Yes	Yes
Individual fixed effect	Yes	Yes	Yes	Yes	Yes
Year fixed effect	Yes	Yes	Yes	Yes	Yes
Urban fixed effect	Yes	Yes	Yes	Yes	Yes
*Obs*	68,575	68,575	43,314	51,544	53,950
*R* ^2^	0.580	0.561	0.493	0.702	0.334

## 6 Channel analysis and selection effect

### 6.1 Channel analysis

The previous analysis suggests that ECER improve health but reduce labor market performance among residents. Existing studies have found that reductions in pollutants are an important cause of improved health (36). Moreover, it has been observed that industrial structural transformation can lead to poorer labor market outcomes for residents, resulting in unemployment or lower wages for workers (37). Considering that ECER policies may reduce regional pollutant emissions and lead to the closure or green transformation of high-energy-consuming and high-polluting industries, this paper aims to test the channels through which ECER influences health and economic effects at macro level. Specifically, we will examine these effects in terms of pollutant reductions and industrial structure transformation.

#### 6.1.1 Pollutant

Pollutants are one of the key factors influencing public health, and air and water pollution are significant sources of environmental contamination in many developing countries (38). High levels of pollutant emissions directly affect residents’ health. Industrial production and other activities generate substantial amounts of smoke, dust, and other emissions, which are more likely to be inhaled and thus damage residents’ health (39). Therefore, reducing pollutant emissions is an effective way to improve human health.

In this study, urban industrial wastewater emissions (*Wastewater*), industrial sulfur dioxide emissions (*SO_2_*), and industrial fumes and dust emissions (Fumes&Dust) are used as explanatory variables in baseline model. As shown in Table 11, ECER demonstration cities significantly reduce pollutant emissions. This indicates that ECER effectively lower pollutant levels. Furthermore, a reduction in pollutant emissions can lead to improved public health (40). Comprehensive analysis reveals that the ECER city pilot policy not only reduces pollutant emissions but also improves residents’ health.

**Table 11:** Channel (*pollutant*).

Variable	*Wastewater*	*SO_2_*	*Fumes*&*Dust*
*ECER*	−1451.470***	−13370.240*	−64572.790***
	(8481.933)	(3333.555)	(23160.770)
Controls	Yes	Yes	Yes
Year fixed effect	Yes	Yes	Yes
Urban fixed effect	Yes	Yes	Yes
*Obs*	2,711	2,988	2,697
*R* ^2^	0.846	0.784	0.206

#### 6.1.2 Structural transformation of industries

Energy consumption in highly polluting and energy-intensive industries is a significant source of pollution emissions and overall energy consumption. Promoting industrial structure transformation is an important approach to achieving ECER goals. ECER city pilot policies emphasize industrial decarbonization, which can lead to the downsizing of high-emission industries or the greening of industrial structures (41). This process may result in enterprise closures or layoffs as part of cost-cutting measures. To explore the influence mechanism of the health effects of ECER, this study estimates the impact of ECER on industrial structure transformation using several proxies: the proportion of added value of the primary, the secondary, the tertiary industry to GDP (*Pri_GDP*; *Sec_GDP*; *Ter_GDP*); the proportion of employees in the primary, the secondary, the tertiary industry (*Pri_Emp*; *Sec_Emp*; *Ter_Emp*). The estimation results, presented in Table 12, show that ECER significantly reduces the proportion of value added of secondary industry to GDP while significantly increasing *Pri_GDP* and *Ter_GDP*. In terms of employment, ECER significantly increases *Pri_Emp* and decreases *Sec_Emp* and *Ter_Emp*. These findings are consistent with (42). The findings suggest that the ECER demonstration city policy promotes the transformation of industries with high energy consumption and significant pollution. Consequently, this shift adversely affects the labor market performance of local residents.

**Table 12:** Channel (*structural transformation of industries*).

Variable	*Pri_GDP*	*Sec_GDP*	*Ter_GDP*	*Pri_Emp*	*Sec_Emp*	*Ter_Emp*
*ECER*	0.695***	−1.547***	0.851*	0.523*	−0.386	−0.107
	(0.258)	(0.533)	(0.443)	(0.289)	(0.700)	(0.708)
Controls	Yes	Yes	Yes	Yes	Yes	Yes
Year fixed effect	Yes	Yes	Yes	Yes	Yes	Yes
Urban fixed effect	Yes	Yes	Yes	Yes	Yes	Yes
*Obs*	2,916	2,916	2,916	2,728	2,777	2,777
*R* ^2^	0.953	0.897	0.916	0.904	0.899	0.883

### 6.2 Selection effect

Based on previous analysis, it is clear that ECER bring benefits such as improved health but also entail costs like declining employment and income. Who gains the benefits, and who bears the costs, which is a critical question for economic equity and social justice. We address the issue by analyzing heterogeneity in macro-regional characteristics (*rural* and *provincial capital*) and micro-demographic characteristics (*chronic* and *exercise*).

#### 6.2.1 Rural and provincial capital

Theoretically, communities in economically underdeveloped areas are more susceptible to the adverse impacts of pollution and labor market dynamics (43, 44). In regions where economic development is limited, pollution levels are often less severe, which means that the health benefits resulting from ECER’s pollution reduction efforts might be less substantial. Furthermore, in these economically disadvantaged areas, the economic distortions caused by ECER’s reductions in production could be more pronounced. Tables 13, 14 report the heterogeneous effects of ECER across macro-regions. It can be observed that the coefficients of ECER affecting population health and labor market performance are more significant in rural areas and non-provincial capital. This finding indicates that both health improvements and economic distortions associated with ECER are more significant in regions characterized by lower economic development, aligning with earlier discussions.

**Table 13:** Heterogeneity analysis (*rural*).

Variable	Health effect			Economic effect	
	*SRH*	*TWDI*	*log*(*ME*)	*ES*	*log*(*Wage*)
Panel A: *City*					
*ECER*	0.090	−0.005*	−0.214	−0.024	−0.109**
	(0.056)	(0.003)	(0.161)	(0.027)	(0.047)
*Obs*	12,884	12,884	7,908	12,797	10,414
*R* ^2^	0.614	0.593	0.539	0.691	0.333
Panel B: *Rural*					
*ECER*	0.070*	−0.004**	−0.203*	−0.042*	−0.020
	(0.038)	(0.002)	(0.106)	(0.024)	(0.034)
*Obs*	50,101	50,101	31,628	33,438	38,872
*R* ^2^	0.571	0.552	0.481	0.701	0.329
Individual characteristics	Yes	Yes	Yes	Yes	Yes
Household characteristics	Yes	Yes	Yes	Yes	Yes
Urban characteristics	Yes	Yes	Yes	Yes	Yes
Individual fixed effect	Yes	Yes	Yes	Yes	Yes
Year fixed effect	Yes	Yes	Yes	Yes	Yes
Urban fixed effect	Yes	Yes	Yes	Yes	Yes

**Table 14:** Heterogeneity analysis (*provincial capital*).

Variable	Health effect			Economic effect	
	*SRH*	*TWDI*	*log*(*ME*)	*ES*	*log*(*Wage*)
Panel A: *Provincial capital*					
*ECER*	0.021	−0.001	−0.281	−0.062*	−0.047
	(0.054)	(0.003)	(0.182)	(0.034)	(0.058)
*Obs*	9,673	9,673	6,113	7,916	7,104
*R* ^2^	0.599	0.562	0.511	0.708	0.339
Panel B: *Other city*					
*ECER*	0.097***	−0.006***	−0.147	−0.073***	−0.053
	(0.038)	(0.002)	(0.104)	(0.020)	(0.032)
*Obs*	58,902	58,902	37,201	43,628	46,846
*R* ^2^	0.578	0.561	0.490	0.701	0.333
Individual characteristics	Yes	Yes	Yes	Yes	Yes
Household characteristics	Yes	Yes	Yes	Yes	Yes
Urban characteristics	Yes	Yes	Yes	Yes	Yes
Individual fixed effect	Yes	Yes	Yes	Yes	Yes
Year fixed effect	Yes	Yes	Yes	Yes	Yes
Urban fixed effect	Yes	Yes	Yes	Yes	Yes

#### 6.2.2 Chronic and exercise

Individuals with compromised physical health or who do not engage in regular exercise are more susceptible to the adverse impacts of pollution and labor market (45). Tables 15, 16 report the heterogeneous effects of ECER based on the presence or absence of chronic diseases or exercise, respectively. It can be observed that both health and economic distortion effect of ECER are more significant in groups with chronic diseases and no exercise. This implies that both the benefits and costs of ECER are experienced by individuals with chronic diseases and no exercise.

**Table 15:** Heterogeneity analysis (*chronic*).

Variable	Health effect			Economic effect	
	*SRH*	*TWDI*	*log*(*ME*)	*WS*	*log*(*Wage*)
Panel A: *Chronic*					
*ECER*	0.069*	−0.004**	−0.110	−0.043**	−0.092**
	(0.039)	(0.002)	(0.104)	(0.020)	(0.030)
*Obs*	48,068	48,068	31,115	37,161	37,431
*R* ^2^	0.570	0.554	0.520	0.709	0.348
Panel B: *No chronic*					
*ECER*	0.049	−0.003	−0.309*	−0.011	−0.017
	(0.057)	(0.002)	(0.161)	(0.043)	(0.055)
*Obs*	14,935	14,935	7,320	9,368	10,994
*R* ^2^	0.589	0.580	0.506	0.716	0.417
Individual characteristics	Yes	Yes	Yes	Yes	Yes
Household characteristics	Yes	Yes	Yes	Yes	Yes
Urban characteristics	Yes	Yes	Yes	Yes	Yes
Individual fixed effect	Yes	Yes	Yes	Yes	Yes
Year fixed effect	Yes	Yes	Yes	Yes	Yes
Urban fixed effect	Yes	Yes	Yes	Yes	Yes

**Table 16:** Heterogeneity analysis (*exercise*).

Variable	Health effect			Economic effect	
	*SRH*	*TWDI*	*log*(*ME*)	*WS*	*log*(*Wage*)
Panel A: *Exercise*					
*ECER*	0.013	−0.001	−0.015	−0.047	−0.073
	(0.060)	(0.003)	(0.163)	(0.037)	(0.053)
*Obs*	40,093	40,093	25,847	33,432	30,452
*R* ^2^	0.655	0.636	0.562	0.754	0.419
Panel B: *No exercise*					
*ECER*	0.661**	−0.032***	−0.848**	−0.180**	−0.067
	(0.217)	(0.012)	(0.422)	(0.086)	(0.185)
*Obs*	1,669	1,669	952	1777	1,259
*R* ^2^	0.715	0.691	0.655	0.770	0.501
Individual characteristics	Yes	Yes	Yes	Yes	Yes
Household characteristics	Yes	Yes	Yes	Yes	Yes
Urban characteristics	Yes	Yes	Yes	Yes	Yes
Individual fixed effect	Yes	Yes	Yes	Yes	Yes
Year fixed effect	Yes	Yes	Yes	Yes	Yes
Urban fixed effect	Yes	Yes	Yes	Yes	Yes

## 7 Welfare analysis of ECER

Based on estimation results presented above and Equation 9, we conservatively estimate average effect of ECER on individual welfare by incorporating a welfare analysis framework (20).

(9) $$\begin{array}{l} \frac{\mathrm{dU}}{\mathrm{dE}}=-\frac{M_{0}}{I-M_{0}}(1+\frac{1}{1+\gamma }\times \frac{I}{I-M_{0}})\times \frac{d\ln M_{0}}{\mathrm{dE}}\\ +\frac{w}{I-M_{0}}(1+\frac{1}{1+\gamma }\times \frac{M_{0}}{I-M_{0}})\times \frac{\mathrm{dl}}{\mathrm{dE}} \end{array}$$

Where *d*ln*U*/*dE* measures the impact of ECER on individual welfare. The first term to the right of Equation 9 indicates the impact of health effect of ECER on individual welfare, which can be calculated based on sample means and regression results in Table 1, columns 5 and 6 from empirical section. The second term to the right of Equation 9 represents the impact of economic effect of ECER on individual welfare, which can be calculated based on sample means and regression results in columns 3 and 4 of Table 2 from empirical section.

Based on Equation 9, we conservatively estimate individual welfare effect of ECER. Here, M0 is expressed as per capita medical expenditure from previous year, which is 3,642.646 yuan; w represents per capita wage income from previous year, which is 28,313.402 yuan; and l indicates per capita employment rate, which is 0.697. Estimation results are shown in Table 17.

**Table 17:** Welfare analysis of ECER.

Parameter	Health effect (>0)	Economic effect (<0)	Total welfare impact(>0)
*γ* = 0	1.822% ~ 2.284%	−1.138% ~ −1.103%	0.684% ~ 1.181%
*γ* = 1	1.029% ~ 1.302%	−0.892% ~ −0.864%	0.137% ~ 0.438%

It can be observed that at individual welfare level, the welfare gains from the reduction of individual medical expenditures due to the health effects of ECER is higher than the welfare loss from the reduction of income. This indicates that ECER is conducive to the improvement of individual welfare levels. Above results imply that residents possess a favorable propensity to remunerate for ECER. The welfare effect of ECER presented here is a conservative estimate. For instance, it underestimates welfare gains from health improvements due to ECER and overestimates the welfare losses from economic distortions caused by ECER (46).

Welfare analysis shows that if environmental pollution is left unchecked and the focus is solely on rapid economic development, the income benefits brought by economic development cannot offset the loss of personal welfare caused by the deterioration of health. In contrast, the health improvements resulting from pollution reduction under ECER and other measures can effectively compensate for welfare loss caused by income reductions. Therefore, ECER and emission reduction efforts have a positive impact on increasing individual welfare levels.

## 8 Limitations and recommendations

This study has several limitations that should be acknowledged. First, the data are derived from CHARLS, which only surveys individuals aged 45 and above. As a result, the findings may not fully capture the health and labor market impacts of ECER on younger populations. Second, although we employed robustness checks such as Heckman two-stage method, potential unobserved confounding factors, such as local governments’ implementation capacity, may still influence the results. Third, we acknowledge that the current questionnaire contains limited objective health measures. Future studies could incorporate more direct and objective indicators of health status, such as clinical examinations or biomarker data, to further validate and extend our findings. Finally, the generalizability of our findings is limited, as the analysis is based on the Chinese context. Caution should be exercised when extrapolating to other countries with different institutional and socio-economic environments.

## 9 Conclusion and policy implications

The consideration of health improvement and economic distortion effects on residents during the process of ECER has emerged as a pivotal issue for achieving sustainable development both in China and globally. Based on the ECER demonstration city policy, we utilized CHARLS database and employed staggered difference-in-differences method to examine the impacts of ECER on residents’ health and labor market performance. Mechanisms and heterogeneity of the impact of the ECER demonstration city on residents’ health and labor market performance are discussed in detail. The welfare effects of ECER are further conservatively estimated from the residents’ perspective by combining the constructed theoretical model.

The results show that ECER significantly improves residents’ health, raises self-rated health (*β* = 0.06, *p* < 0.05, 95% CI = −0.17 to 0.13), reduces the probability of illness affecting work (*β* = −0.004, *p* < 0.05, 95% CI = −0.01 to 0.01), and lowers medical expenditures (*β* = −0.183, *p* < 0.05, 95% CI = −0.64 to 0.10). However, ECER negatively affects residents’ labor market performance, reducing employment status (*β* = −0.032, *p* < 0.10, 95% CI = −0.11 to 0.06) and wage (*β* = −0.055, *p* < 0.05, 95% CI = −0.23 to 0.00). This conclusion remains consistent after robustness checks, including event study, PSM-DID estimation, placebo tests, excluding the interaction between economic and health effects, excluding sample selection bias, excluding self-selection bias, excluding contemporaneous policy interference, and altering the level of standard error clustering. Mechanism analysis reveals that ECER can reduce pollutant emissions (such as industrial sulfur dioxide emissions and industrial smoke and dust emissions), thereby improving residents’ health. ECER promotes the transformation of industrial structure (e.g., reducing the proportion of value-added of the secondary industry in GDP and the proportion of employees in the secondary industry), which impacts residents’ labor market performance. Heterogeneity analysis shows that there is a selection effect in the impact, with the health benefits and economic costs of ECER mostly obtained and borne by groups in rural areas, non-provincial capital cities, and those with chronic diseases and no exercise. Welfare analysis shows that the health benefits of ECER result in a higher welfare increase than the economic costs, leading to an overall net welfare gain.

Three key policy recommendations emerge from this study. First, the introduction of ECER demonstration city policy has significantly improved residents’ health and welfare, providing a strong rationale for scaling up similar initiatives both within China and internationally. Second, the study found that there is heterogeneity in the extent to which ECER affects health and employment at the macro and micro levels, and therefore future policy frameworks should take into account differences between groups with different health and employment status and pay more attention to disadvantaged groups. Third, welfare analysis has shown that ECERs negatively impact labour market dynamics while improving overall health and well-being. Future policy should therefore take a balanced approach to assessing the pros and cons, with a focus on mitigating the loss of well-being for those who bear the higher costs.

## Data Availability

The datasets presented in this study can be found in online repositories. The names of the repository/repositories and accession number(s) can be found at: https://charls.pku.edu.cn/.
